# Hospital Meetings

**Published:** 1906-06-02

**Authors:** 


### HOSPITAL MEETINGS.

### BROMPTON HOSPITAL FOR CONSUMPTION.

#### A SAVING OF £20 PER BED REPORTED.

Major-General Lord Cheylesmore, Chairman of the Committee of Management, presided on Friday, May 25, at the sixty-fifth annual Court of Governors of the Hospital for Consumption and Diseases of the Chest, Brompton, in the board-room of the hospital.

Lord Cheylesmore read the report of the Committee, which stated that there were admitted during 1905 1,529 patients, of whom 1,232 were discharged, many greatly benefited, 206 were transferred to the sanatorium and convalescent home, and 101 died. There were in the hospital on December 31 307 patients. The daily average of beds occupied was 313, the average stay of the patients being 74.7 days. The number of new out-patient cases was 11,966, and the attendances 61,744. The amount received from subscriptions had been fairly well maintained, but the donations and legacies were somewhat smaller; £1,000 was received from the King's Fund, and a donation of £525 from Mrs. Isaac Feldheim to name a memorial ward; £9,550 was the total of the legacies announced since the last court. The sanatorium had now been in full working order for nearly a year, and the report from the medical staff showed most satisfactory results from the treatment. While thanking those who had made presents of books, flowers, games, etc., the Committee pointed out that since the sanatorium had been opened there was an increased demand for such gifts, which were always very acceptable. On the recommendation of the medical staff and advice of the consulting engineer, an entirely new steam sterilising plant had been established for dealing with sputum, and a small washing-machine and callender for handkerchiefs, of which a clean one was given to each phthisical patient every day. Part of the hospital buildings had been insured in the Westminster Fire Office, and now that that office had been absorbed by the Alliance Insurance Company, the hospital had received as its share in the scheme a cheque for £1,012 and a free insurance policy for twenty-eight years. Favourable offers for the supply of electric current having been secured, the Committee had decided to sell the existing plant, and take current from a public supply company. With regard to the financial position, the Committee regretted that, consequent on the opening of the sanatorium and convalescent home, and in spite of great economies and heavy retrenchments during the past two years, amounting to a saving of no less than £20 per bed, the year's working still showed a deficit of £5,000, or £7,000 with the extraordinary expenditure. They therefore appealed urgently for further help, so that it might not be found necessary to consider the possibility of having to close a portion of the hospital at some early date, which would mean that not only could a smaller number of patients be treated, but that those who were admitted would have to wait much longer than at present for admission, during which period of waiting it was often found that their condition became rapidly worse.

The report was adopted and the Committee re-elected, and votes of thanks to the medical officers and to the Chairman brought the proceedings to a close.

### HOSPITAL FOR SICK CHILDREN.

The Duke of Fife, President of the hospital, took the chair on the occasion of the annual Court of Governors on May 25. In moving the adoption of the report the Duke of Fife remarked that the eternal financial difficulty was always staring them in the face. The voluntary system of maintenance of the hospitals, which he would be very sorry to see disappear, was now on its trial, and unless the London public would support the hospitals more liberally he greatly feared that the system would stand condemned. It was no use saying that this or that fund for the maintenance of hospitals had increased so much and there was more money given now than a quarter of a century ago. The question was whether the contributions had increased in proportion to the increase in population. This was the first children's hospital started in the United Kingdom, and it had served as a pioneer and a model for all other children's hospitals. It might also claim to have led the way to a greater interest being taken in children, in their health and general well-being. They heard now of societies being established to teach mothers how to feed their babies and to teach children how to play. This hospital had led the way in this movement, which was one of the distinctive features of the day. Nowhere would the study of children's diseases be better carried than there, owing to its great size, and it offered a wide and varied field of observation to students from the general hospitals who were always welcomed there.

The motion was seconded by Mr. Arthur Lucas, Chairman of the Committee, who also moved that the proceedings of the Committee of Management during the past year should be confirmed. Their total expenditure, he said, was £18,929, a slight increase over that of the previous year, but they had treated 336 more patients, so that the increase was more than justified. The average cost of the stay of each patient was £4 16s. 4d., as against £5 5s. 1d. in 1904. Great credit for this decrease was due to the matron and housekeeper. The increase in the number of out-patients was only 126, and as 37,000 were treated the increase could not be considered large. They had appointed a casualty medical officer who saw every patient, but no patient brought there left the hospital without being seen by one of the medical or surgical staff, so as to avoid all risk of danger. With regard to Mr. Astor's generous gift for the new out-patients' department, they had not been able to obtain possession of the site till last autumn, but the plans were all now prepared and they hoped by the end of June the contracts would be placed, and they would then be able to begin building immediately.

Mr. John Murray seconded the resolution, which was carried, as were various others, including a vote of thanks to the Duke of Fife for taking the chair.

### PADDINGTON GREEN CHILDREN'S HOSPITAL.

The annual meeting of Governors of this hospital was held on May 24, Mr. Douglas Owen, Chairman of the Committee, presiding. The adoption of the annual report and balance-sheet was moved by Mr. George Hanbury, Treasurer, who remarked that in this hospital they did an immense deal towards the alleviation of suffering, and many children after treatment there went to a convalescent home or back to their own homes, and grew up by degrees strong and well. It was said sometimes that people sent their children to the hospital who ought to pay for them, but it was quite impossible for the children to be as well cared for in their own homes, and the parents were most thankful for the blessing the Children's Hospital was to them. They were doing a very good work; they had a most excellent matron and attentive nurses, and everything possible was done towards the recovery of the children, who were many of them as happy as could be.

Mr. W. Bury seconded the motion, and remarked that he hoped in a few days, in conjunction with a friend, to sign a cheque which would relieve the minds of the Committee a good deal as to their financial position.

A vote of thanks was proposed by the Chairman to the members of the honorary medical and surgical staff. He had been a member of the Committee for twenty-two years, and he was of opinion that the Medical Committee left nothing to be desired; they offered the utmost assistance on all occasions, and they were as reasonable as they could be in their demands for new implements, etc. If the Committee were not able to meet all their requests (which was very rarely) they accepted the fact cheerfully. They were, in short, quite beyond praise. He wished also to include in this vote of thanks the names of the matron and the Secretary. It was with very great satisfaction he heard Mr. Bury state that he was about to sign a cheque, and he hoped it would be for a good amount. The sum given by King Edward's Hospital Fund was double what it had been the year before, and it was encouraging to conclude from that that the King's Fund thought they were doing good work. They were all immensely grateful to the lady visitors for great kindness to the little patients. They were hoping to start a Ladies' Association in connection with the hospital.

The vote was seconded by Mr. C. W. Empson, and carried.

Dr. Keogh Murphy, in responding to the vote, drew attention to the large increase in attendances at the out-patients' department, the figures being 58,037 in 1905, as against 50,500 in 1903. It was a lamentable fact that the beds in convalescent homes were far too few to take in all the children who required a stay there. Many children, in particular surgical cases, did not need treatment so much as two or three weeks in good country air to restore them to health before returning to their own homes. He threw this out as a suggestion to the ladies present. Perhaps some of them had houses in the country or old servants living in cottages who might take in some of these children for a few weeks.

A vote of thanks to the Chairman terminated the proceedings.

### VICTORIA HOSPITAL FOR CHILDREN, CHELSEA.

The annual meeting of Governors of this hospital was held on May 16. The chair was taken by the Hon. R. A. Lubbock, who, in moving the adoption of the report, said that it was his proud privilege to be able to record a further signal mark of royal favour from her Majesty Queen Alexandra. On July 22, 1905, her Majesty paid a private visit to the hospital, and was then graciously pleased to sanction their naming one of the vacant wards the "Alexandra" ward, in commemoration of her visit. For some reasons they might perhaps have wished that this visit had been a little less informal, but the evident interest which her Majesty showed in all the arrangements and the entire approval which she was graciously pleased to express could not but gratify all those who, having the interests of the Victoria Hospital entirely at heart, had endeavoured to make it rank amongst the most up-to-date institutions for the relief of suffering.

Whilst they could not but feel satisfied that their efforts had resulted in the erection of their new buildings and the remodelling of the old at a cost of £50,000, and that they would shortly be in a position to reduce their debt to the bankers to below £3,000, they were faced with the fact that their annual expenditure had increased, and it would be necessary for them to make special efforts to obtain sufficient extra support from the public to raise the sum of £2,000, which would be necessary every year if they were to keep out of debt and maintain the hospital in a thoroughly efficient manner. He expressed his regret once more that they did not receive more local support, though a pleasing feature was the formation of trade cots, a scheme suggested by the Secretary, which seemed to be meeting with considerable success.

The Convalescent Home at Broadstairs continued to be a very useful adjunct to the hospital, but there, again, they sadly lacked financial support, though he was pleased to see that many of their subscribers were adding a little to their usual subscription for this essential branch of the work.

The chief feature of the work in 1905 was the opening of the two wards which they had been obliged to keep closed for more than a year for want of funds, and it was mainly due to the kind and generous interest of Mr. Gardner and his co-trustees that this had been accomplished. As a result they had been able to do a greater amount of work in the in-patient department, and at a proportionately decreased cost. The cost per occupied bed in 1905 was 29s. 8d., as against 35s. 6d. in 1904, which brought them more into line with other children's hospitals, and should prove to the authorities of the great hospital funds that they were doing their best to work as economically as efficiency would permit.

After paying a tribute to the excellent work done by the Secretary and the matron, he acknowledged with thanks the services which the honorary medical officers had so ably rendered to the hospital and for the help they gave the committee.

The motion was seconded by Dr. Ridge Jones and carried.

Mr. D'Arcy Power, F.R.C.S., having resigned his position as surgeon to in-patients after a service of nineteen years, was subsequently elected a consulting surgeon.

### CONVALESCENT HOMES ASSOCIATION.

Sir William Church, M.D., presided on Thursday, May 17, at the first annual meeting of the Council of the Convalescent Homes Association. The Association was formally constituted last October, its objects being to coordinate the work of public convalescent homes receiving London convalescent patients, whether from their own homes or from the Metropolitan hospitals, such homes being eligible to become members of the Association; to obtain and disseminate information on the needs of London in respect of convalescent relief and on all other matters of interest or importance to members; to advise them, if requested, on equipment, management, and maintenance; to watch over and protect their rights and interests as affected by legislation or otherwise; to serve as a means of communication and to assist co-operation between convalescent homes and hospitals and other public institutions; and generally to take such action as may be desirable in any matter in which convalescent homes are interested.

Dr. Habershon, one of the Hon. Secretaries, read the report of the Executive Committee of the first six months' working of the Association, as follows :—

"Fifteen homes have become members and five hospitals have joined (St. Bartholomew's, St. Thomas's, King's College, the Royal Free, and the Poplar Hospitals). While not intending to create a new fund for the aid of convalescent institutions, the Association has from the outset been hampered by the want of funds for ordinary working expenses. It is impossible to carry out the intentions of the Council and the Executive Committee to the fullest degree unless sufficient homes and hospitals join and pay the small subscription desired. An office and secretary have been temporarily provided, through the courtesy and consideration of the Metropolitan Convalescent Institution. It is intended to open a central bureau to provide information to hospitals and to the public on convalescent matters, and to provide beds at existing homes for special cases. This plan is not possible until we can provide a separate office and secretary. The subscriptions of the various homes and hospitals which have joined will barely reach £40. For the full current expenses of the Association about £150 would be required per annum. In spite of these discouragements the efforts of the Convalescent Homes Association have been already attended with some measure of success, and the Committee is not without hope that as the Association gradually gets to work, both homes and hospitals will be convinced of the advantage of joining. The initial work has been to endeavour to meet the needs of hospitals by opening up special beds for patients recovering from operations with unhealed wounds. Some forty additional beds have already been established for these purposes. The Metropolitan Convalescent Institution has inaugurated twenty new surgical beds, ten for men and ten for women. Mrs. Gladstone's Convalescent Home will shortly be in a position to offer nine beds for male surgical cases. Mrs. Bischoffsheim offers five beds (three for men and two for women) at the Baroness de Hirsch's Convalescent Home for Jews. Mrs. Spender offers one bed for children, and the Hon. Mrs. Campion, at the Sunshine Home, will provide one or two others. Moreover, an effort is being made to open up more special surgical beds for children, and the Committee hope that in future much may be done in this direction. The Committee propose to ask the members of the Association to furnish them (in confidence) with a copy of their "Black List," with a view to excluding undesirable and regular applicants. The Committee hope also shortly to consider a scheme for establishing a register of beds vacant at affiliated homes from time to time to enable hospitals to ascertain on application where patients can be most promptly and suitably treated during their convalescence. They also hope that in future the affiliated hospitals may be induced to give preference to those convalescent homes which are members of the Association."

This report Dr. Habershon said might, he thought, be considered very satisfactory for their initial period. They were moving in various directions, one of them being a proposal to the L.C.C. for the use of some of its mansions for convalescent home purposes. At Golder's Hill in particular they were trying, but were meeting with some difficulty with the Hampstead Borough Council, which was not favourable to the idea.

On the motion of Sir Thomas Smith, seconded by Miss Alice Gladstone, the report was adopted, as was also a motion that Lord Lytton, Sir Henry Harben, Mr. Peter Reid, and Mr. A. F. Yarrow be made vice-presidents.

On the motion of the Chairman it was resolved that the present Executive Committee be continued in office, and after considerable informal conversation concerning various matters connected with the objects of the Association, the meeting separated with the customary votes of thanks.

### PREMATURE BURIAL.

A well-attended meeting of the London Association for the Prevention of Premature Burial was held at Bloomsbury Hall on Thursday, May 24, Mr. George Greenwood, M.P., presiding. In opening the proceedings the Chairman said the subject was a gruesome one, and many people were repelled by it, but it was of vital importance. We should not ask what were the chances of being buried alive, but was it possible? If it was, the idea was so horrible that the need for legislation was established. Even the most cursory glance at the evidence obtainable must show that this was so. It was matter of common knowledge that the law with regard to death certificates was a perfect scandal. The Association was increasing in numbers, which showed that people were awakening to the realisation of the dangers it was their object to combat.

Dr. Stenson Hooker bore testimony to the increasing interest taken in the subject. It was a prevalent custom for directions to be given in wills that some mutilation of the body should take place to make sure of the fact of death.

It would be much simpler and less repugnant if people would join that Society, and so ensure that their bodies would be watched until there was no possibility of live burial. He considered that post-mortems took place too soon after apparent death. It occurred frequently at inquests that the post-mortem examination revealed no cause of death. It was an anomaly of medical training that students went through their whole curriculum frequently without a single question arising as to the signs of death.

Dr. Walter Hadwen gave instances from the second edition of "Premature Burial and how it may be Prevented," which he had just edited, to show that the danger was a real one. He quoted from the observations of a coroner in 1902 at an inquest on a child which had apparently died four times, three medical certificates of death having been given on the mother's diagnosis. In the compilation of the book thousands of instances had been brought before them, but excluding practically all those not verified by medical men there remained 384, of which 149 were buried alive, 219 had narrow escapes from it; 10 were dissected alive and 3 narrowly escaped it; 2 were embalmed alive and 1 was burned alive. These, he said, were 384 solid arguments in favour of speedy legislation for burial reform. Only two of every 100,000 bodies were exhumed in the United Kingdom. Mistakes were buried out of sight, and the coffin boards were the sole witnesses of the tragedy. He urged that waiting mortuaries should be erected all over the country, similar to those in Munich and other parts of Germany, which he described in detail. The bodies of the poor especially should be removed from their cramped surroundings to pleasant and well-ventilated mortuaries decorated with flowers, where friends could visit them, and there be left under strictly sanitary conditions until the first sign of decomposition had set in, for, he said, no single sign or combination of signs could be considered satisfactory apart from this. A finger of the supposed corpse should be placed in a ring delicately adjusted in connection with an electric bell communicating with a room in which an attendant would be present day and night, so that the slightest movement would sound the alarm and bring the attendant immediately upon the spot. The expense would not be more than could be met by a rate of from one farthing to a penny in the pound, and in the smaller and thinly-populated districts groups of parishes could unite together to provide such institutions. The mortuaries of London were for the most part a scandal to a great municipality, and the provincial establishments were, if in existence, in many instances worse. There were ten magnificent mortuaries in Munich, and burial alive was a practical impossibility. Not only did a person stand the risk of being buried alive in this country, even when a medical certificate of death had been granted, as proved by several recent instances, but it was a fact that thousands of persons were buried every year without even a medical certificate having been granted at all; and no medical man was under any obligation to see a dead body before he granted it.

Miss Lind-af-Hageby, Mr. Alexander Johnson, and Archdeacon Colley also addressed the meeting, and Dr. Brimley James moved the following resolution: "That this meeting heartily approves, and pledges itself to strenuously support, the draft Bill of the L.A.P.P.B., which provides that no death certificate shall be given without a personal inspection and examination of a supposed dead body by a qualified officer appointed for the purpose, and that waiting mortuaries shall be established for the reception and detention of doubtful cases until the fact of death has been conclusively ascertained." This was agreed to, and the proceedings terminated.

